# Bone Suppression Increases the Visibility of Invasive Pulmonary Aspergillosis in Chest Radiographs

**DOI:** 10.1371/journal.pone.0108551

**Published:** 2014-10-03

**Authors:** Steven Schalekamp, Bram van Ginneken, Inge A. H. van den Berk, Ieneke J. C. Hartmann, Miranda M. Snoeren, Arlette E. Odink, Winnifred van Lankeren, Sjoert A. H. Pegge, Laura J. Schijf, Nico Karssemeijer, Cornelia M. Schaefer-Prokop

**Affiliations:** 1 Dept. Of Radiology, Radboud University Medical Center, Nijmegen, The Netherlands; 2 Dept. Of Radiology, Meander Medical Center, Amersfoort, The Netherlands; 3 Dept. Of Radiology, Academic Medical Center, Amsterdam, The Netherlands; 4 Dept. Of Radiology, Maasstad Hospital, Rotterdam, The Netherlands; 5 Dept. Of Radiology, Erasmus Medical Center, Rotterdam, The Netherlands; The University of Hong Kong, Hong Kong

## Abstract

**Objective:**

Chest radiographs (CXR) are an important diagnostic tool for the detection of invasive pulmonary aspergillosis (IPA) in critically ill patients, but their diagnostic value is limited by a poor sensitivity. By using advanced image processing, the aim of this study was to increase the value of chest radiographs in the diagnostic work up of neutropenic patients who are suspected of IPA.

**Methods:**

The frontal CXRs of 105 suspected cases of IPA were collected from four institutions. Radiographs could contain single or multiple sites of infection. CT was used as reference standard. Five radiologists and two residents participated in an observer study for the detection of IPA on CXRs with and without bone suppressed images (ClearRead BSI 3.2; Riverain Technologies). The evaluation was performed separately for the right and left lung, resulting in 78 diseased cases (or lungs) and 132 normal cases (or lungs). For each image, observers scored the likelihood of focal infectious lesions being present on a continuous scale (0–100). The area under the receiver operating characteristics curve (AUC) served as the performance measure. Sensitivity and specificity were calculated by considering only the lungs with a suspiciousness score of greater than 50 to be positive.

**Results:**

The average AUC for only CXRs was 0.815. Performance significantly increased, to 0.853, when evaluation was aided with BSI (p = 0.01). Sensitivity increased from 49% to 66% with BSI, while specificity decreased from 95% to 90%.

**Conclusion:**

The detection of IPA in CXRs can be improved when their evaluation is aided by bone suppressed images. BSI improved the sensitivity of the CXR examination, outweighing a small loss in specificity.

## Introduction

Invasive pulmonary aspergillosis (IPA) is a major cause of morbidity and mortality, particularly in patients with hematological malignancies [1]–[3]. A delay in treatment is associated with substantially increased hospital mortality [4]–[7]. However, treatment is expensive, and can have serious side effects. From a clinical point of view there is therefore a strong need for prompt recognition and proper onset of treatment to ensure an optimal patient outcome.

Current EORTC/MSG (European Organization for Research and Treatment of Cancer, US Mycoses Study Group) guidelines differentiate three levels of probability for the presence of an invasive fungal infection: proven, probable or possible [8]. While a proven diagnosis requires histological and/or cultural evidence from tissue specimen or positive cultures from body fluids, these criteria are often not met and not applicable at an early stage of infection. This discrepancy between definite diagnostic proof of IPA on one side and the need for treatment as early as possible on the other side has led to different therapeutic strategies ranging from prophylaxis to empirical therapy of persistent neutropenia to pre-emptive approaches of very early, still preclinical disease [9]. In this diagnostic dilemma, imaging still plays an important role. In common with other lung diseases, Computed Tomography (CT) has been proven to be more sensitive than radiography in revealing subtle pathology [10]. As consequence, the imaging criteria of the EORTC/MSG guidelines for the presence of IPA refer to specific CT findings (dense well circumscribed lesions with or without a halo sign, air crescent sign, cavity) and also the guidelines published by the Infectious Diseases Working Party of the German Society of Haematology and Oncology (AGIHO) in 2012 state that in patients with granulocytopenia, high resolution computed tomography (HRCT) should be preferred to chest x-ray for primary diagnosis in high-risk patients [11].

Though CT is currently recommended as the preferred method if there is strong clinical suspicion for an infection, chest x-ray (CXR) remains the first line imaging method because of its immediate availability and lower organizational demands. Chest X-rays are also performed if the clinical suspicion is low and/or clinical symptoms are less obvious or not yet fully developed. Also, guidelines by the American College of Radiology [12] and the Infectious Disease Society of America [13] mention chest radiography as first line method for the work up of a patient with neutropenic fever, but do emphasize the low sensitivity of CXR. Thus, chest radiography is still widely applied despite its known diagnostic limitations, meaning an increased sensitivity for the detection of subtle infiltrations would represent a desirable diagnostic improvement. Early recognition of IPA on CXR would trigger an immediate CT examination of the patient in order to render morphology and accelerate the diagnosis, providing a chance to hasten the onset of treatment.

To improve the detection of IPA on CXR, we tested an advanced image processing solution called bone suppression imaging (BSI). This software solution suppresses bone structures in chest x-rays, which can obscure abnormalities. No special hardware or additional dose for the patient is needed. The processing can be applied to both upright and bedside radiographs using imaging equipment of varying manufacturers.

BSI has been shown to be helpful in the detection of lung nodules and focal pneumonia [14]–[16]. We believe this means it might also provide an opportunity to improve the detection of IPA. We therefore decided to undertake an observer study to test the effect of BSI on the detection of IPA in neutropenic patients. If BSI would improve detection of IPA, and so possibly avoid delay in treatment, it could result in an optimized use of CXR in immunocompromised patients at risk of developing IPA.

## Methods

The selection of study images and study setup were waived by the institutional review board (Commissie Mensgebonden Onderzoek regio Arnhem – Nijmegen; www.cmoregio-a-n.nl; nr. 2011/468). Written informed consent was also waived by the institutional review board.

### Image selection

Neutropenic patients with typical signs of invasive pulmonary aspergillosis infection on CT, which had a CXR at a time near the CT scan were selected. Study images were retrospectively retrieved from the image archives of four hospitals (three academic and one non-academic hospital) and anonymized. All images had been obtained for clinical purposes. Patients could have multiple sites of disease per lung, but patients with diffuse lung disease, or large obvious consolidations were excluded. The diagnoses of invasive aspergillus as an underlying disease were taken from the clinical patient charts and included the presence of typical CT findings in all diseased patients. Neutropenic patients with signs of fever with a normal chest radiograph functioned as control subjects. The absence of disease in these control subjects was confirmed by a negative CT after the CXR. Both upright as well as bedside acquisitions were included.

**Figure 1:**
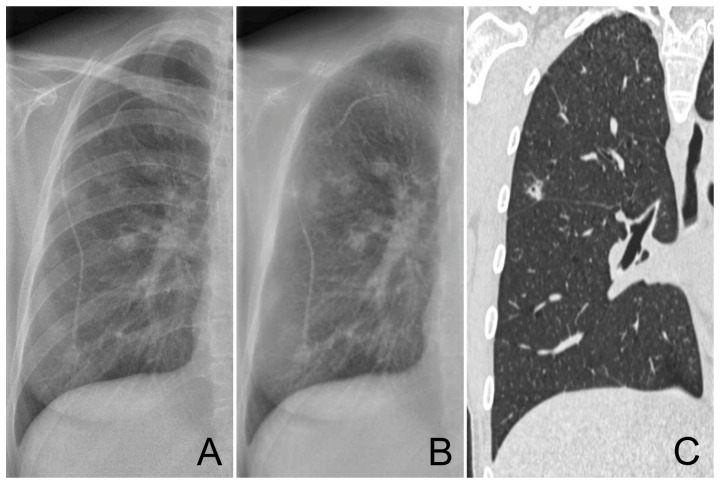
Example of a moderately subtle case of a 35 year old female with multiple focal lesions in the right lung. Without BSI 6 of the 7 observers marked this case with an average suspiciousness score of 63. With BSI all observers marked the case with an average suspiciousness score of 91. Original radiograph (A), bone suppressed image (B), coronal CT slice (C).

The visibility of disease was retrospectively classified based on the suspiciousness scores assigned by the readers participating in the study. Cases that were assigned an average score above 75 (0 = not suspicious, 100 = very suspicious) were classified as *obvious cases* (Figure 1). Cases with average scores between 50 and 75 were classified as *moderately subtle cases*. Cases with an average score below 50 but higher than 25 were labeled as *subtle cases*. If the average score of a case was below 25 then the case was considered *very subtle*.

### Image acquisition

All chest radiographs were obtained with a digital technique using storage phosphor plates (CR, Agfa, Mortsel Belgium), a Selenium drum (Thoravision, Philips, Hamburg Germany) and a flat panel detector DR systems (Siemens, Erlangen, Germany). Bedside images were acquired with the same DR or CR systems. Image processing was applied as recommended by the manufacturers and as used in clinical routine.

### Software

Bone suppressed images were generated by ClearRead Bone Suppression 3.2, from Riverain Technologies (Miamisburg, Ohio). This visualization software uses advanced image processing techniques to construct a bone suppressed frontal chest image. Not only bones obscuring the lung fields are being suppressed, but also other skeletal structures superprojecting the lungs such as the claviculae or the scapulae are eliminated. The software product has U.S. Food and Drug Administration (FDA) approval. Since BSI is a post-processing technique, no special hardware is needed and no additional radiation dose is administered to the patient. BSI is applicable to any digital radiographic chest technique, irrespective of whether the images were obtained at the bedside or in the upright position.

After acquisition of the radiograph, data processing is performed automatically in the background. The processed images are made available in the Picture Archiving and Communication System (PACS) system. The bone suppressed images are not designed to replace the original images but rather to serve as supplement to the original frontal chest radiograph. Both the original and bone suppressed image can be visualized side by side or as stack of two images. Both images are equivalent in terms of size and gradation characteristics, with the exception of overlying rib structures.

### Observer study

Five radiologists (ranging from 3–17 years of experience) and two radiology residents (3^rd^ and 5^th^ year of residency) read all cases during one sequential reading session. Evaluation was carried out separately for the right and left lung, resulting in 210 images of 105 patients. Radiologists were asked to score the most suspicious lesion per image, if lesions were seen. Images on which the observer did not detect a focal lesion received no score. Scores were given on a scale from 1 to 100. There were no instructions how to use this scoring scale, but the observers were motivated to use the full range of scores of suspicion (1–100). Observers first provided a score for the standard CXR, followed by a second score for the evaluation of the standard radiograph supplemented by the bone suppressed image. Observers were able to add or (re)move marks and adjust their scores. No clinical information, besides the knowledge that it was a neutropenic patient group, was provided to the readers. A training set of 40 lungs was provided in advance, containing pulmonary focal opacities of varying conspicuity to make the observers familiar with the appearance of the processed images.

### Statistics

Observer performance was measured using Multi Reader Multi Case (MRMC) receiver operating characteristic (ROC) analysis. ROC analysis calculates the readers' sensitivity as a function of specificity using the assigned ratings per case (suspiciousness scores). The area under the ROC curve (AUC) summarizes the performance of the reader, representing the chance that a positive (diseased) case will be scored with more suspicion than a negative (normal) case. Per reader, each case was assigned the score (1–100) of the most suspicious finding, if present. Differences in observer performance were tested using the Dorfman, Berbaum and Metz method (DBM MRMC package v2.33) which includes case, reader and treatment variance into analysis [17]–[19]. Non-parametric statistical test was used to generate ROC curves and calculate p-values. Besides ROC analysis, sensitivities and specificities were computed for individual observers considering markings exceeding a confidence score of 50.

## Results

### Patient demographics

In total 105 patients were selected. In this group there were 78 diseased lungs and 132 normal lungs. The average age of patients with diseased lungs was 53.8 years and average age of patients with normal lungs was 43.0. On average, CT scans were made 2.6 days after the chest radiographs for diseased patients, compared to 3.7 days for patients with normal lungs. Patient characteristics are summarized in Table 1. Radiographs of 128 lungs were obtained upright, and 82 lungs were acquired at the bedside. With respect to the conspicuity of pathology, diseased lungs were classified as being obvious in 17, moderately subtle in 21, subtle in 15, and very subtle in 25 cases (Table 2).

**Table 1:** Patient demographics.

	Diseased cases (n = 78)	Normal cases (n = 132)
Age	53.8 (range 16–83)	43.0 (range 8–76)
Male	47 (60%)	87 (66%)
Female	31 (40%)	45 (34%)
Days between CXR and CT (median)	2.6 (2)	3.7 (3)
Bedside	22 (28%)	60 (45%)
Upright	56 (72%)	72 (55%)

Age, gender, average time between the chest radiograph and the CT in days, and projection type are displayed for the diseased and normal cases. n  =  number.

**Table 2:** Subtlety categories.

	Bedside	Upright	Total
Obvious	4	13	17
Moderately subtle	8	13	21
Subtle	3	12	15
Very subtle	7	18	25
**Total**	**22**	**56**	**78**

Number of cases classified into the different subtlety categories.

### Observer performance: ROC analysis

The average AUC without BSI was 0.815 against 0.853 with BSI (p = 0.01). Six of the seven observers improved their performance (Table 3).

**Table 3:** Observer performance.

	AUC		
	*Without BSI*	*With BSI*	P
*All images* (n = 210)	0.815	0.853	0.01
*Upright images* (n = 128)	0.804	0.853	0.02
*Bedside images* (n = 82)	0.838	0.878	0.15

Average area under the ROC curves (AUC) for all observers. AUCs are displayed for all images and for analysis of the different groups of projection type (bedside and upright).

**Figure 2:**
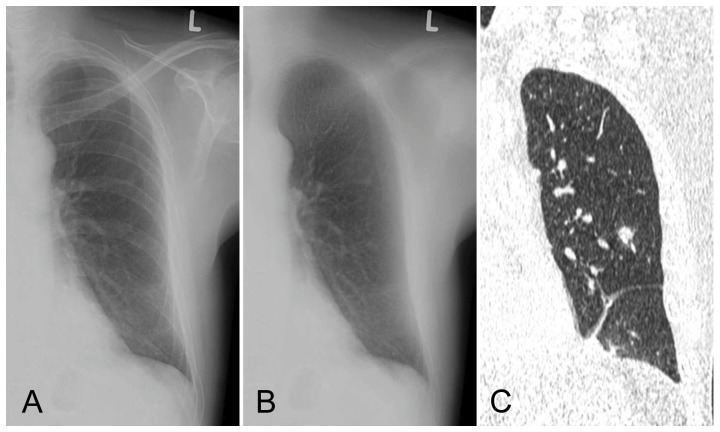
Upright radiograph with a very subtle lesion in the left lower lobe. Without BSI only one observer marked the small lesion. With BSI all 7 observers marked the lesion with an average suspiciousness score of 55. Original radiograph (A), bone suppressed image (B), coronal CT slice (C).

**Figure 3:**
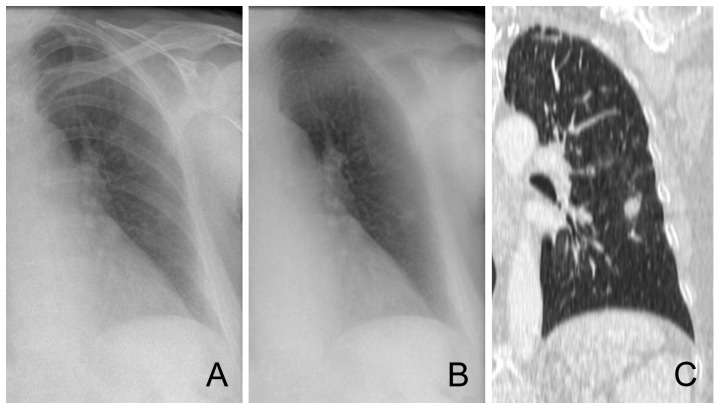
Bedside radiograph of a 58 year old female with neutropenic fever. Without BSI, 3 of the 7 observers called the radiograph suspicious, which increased to 7 observers with BSI. Original radiograph (A), bone suppressed image (B), coronal CT slice (C).

ROC analysis for the different subsets of subtlety classification based on reader scores showed that the very subtle cases benefitted the most from BSI. The AUC for very subtle cases increased from 0.579 to 0.659 (p = 0.02), AUC for subtle cases increased from 0.809 to 0.856 (p = 0.07). For the detection of moderately subtle and obvious cases baseline performance was already very good (AUC = 0.954 and 0.995, respectively) and did not significantly increase with BSI (0.968 and 0.992, p = 0.26 and p = 0.44, respectively). Examples of cases are illustrated in Figures 1–3.

### Analysis of type of projection

The average area under the curve for upright radiographs (n = 128) was 0.804 and significantly increased to 0.853 with BSI (p = 0.02). For bedside images (n = 82) similar improvement was seen with an AUC increasing from 0.838 to 0.878, although the difference did not reach statistical significance (p = 0.15) (Table 3).

### Observer performance: Sensitivity and specificity

**Figure 4:**
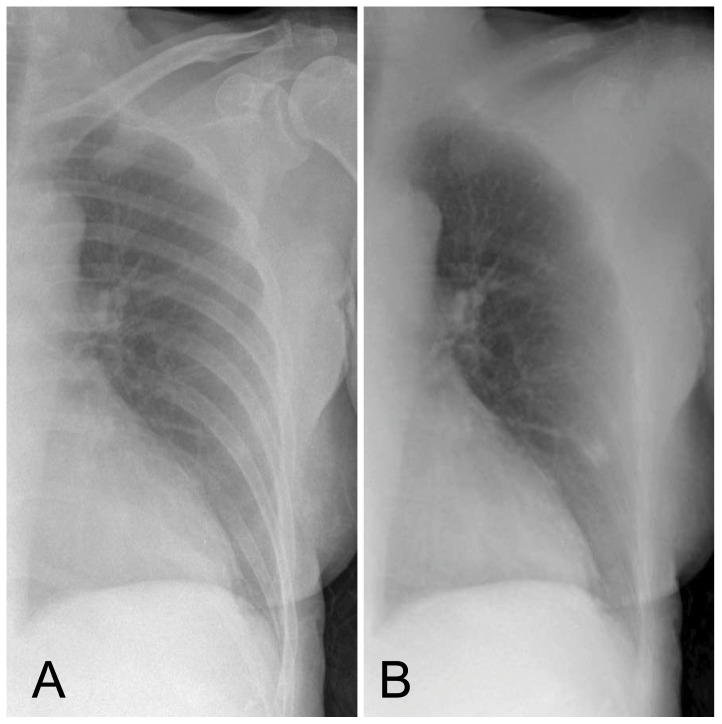
Example of a bedside case that was rated false positive by all 7 observers. Due to an incomplete suppression of the rib crossing of the anterior contour of the 5^th^ rib and the posterior contour of the 8^th^ rib on the BSI, the observers rated the shadow as infectious rounded opacity. Original radiograph (A), bone suppressed image (B).

Overall sensitivity for the detection of IPA was 49 percent without BSI. With the availability of BSI, sensitivity increased to 66%. This resulted, on average, in an additional detection of 13 diseased cases (range 1–38), especially in the more subtle cases (Table 4). Specificity showed a decrease from 95% without BSI to 90% with BSI. On average, 6 normal cases (range 0–13) were marked without BSI, and 14 normal cases (range 2–23) with BSI. Individual sensitivities and specificities are displayed in Table 5. The lower specificity was mainly caused by marking of pseudolesions caused by artifacts of the bone suppression technique (Figure 4).

**Table 4:** Number of, on average, detected cases per subtlety category.

	Without BSI	With BSI
Obvious (n = 17)	16	17
Moderately subtle (n = 21)	15	18
Subtle (n = 15)	6	9
Very subtle (n = 25)	2	8
**Total (n = 78)**	**39**	**52**

**Table 5:** Sensitivity and specificity.

	Sensitivity			Specificity		
Observer	Without BSI (%)	With BSI (%)	Δ (%)*	Without BSI (%)	With BSI (%)	Δ (%)*
Rad 1	44	62	+18	97	91	−6
Rad 2	42	44	+2	98	98	0
Rad 3	62	68	+6	92	87	−5
Rad 4	42	73	+31	97	86	−11
Res 5	15	64	+49	100	91	−9
Res 6	64	69	+5	92	92	0
Rad 7	72	82	+10	90	83	−7
**Average**	**49**	**66**	**+17**	**95**	**90**	**−5**

Individual sensitivities and specificities for the detection of IPA in chest radiographs without and with BSI, based on marking with a suspiciousness score above 50. * Δ = difference.

## Discussion

The results of our study indicate a significantly improved detection of focal opacities due to fungal infection, when supported by bone subtracted images. This improvement was especially striking for very subtle lesions, that otherwise would not be reported. With this new processing, the sensitivity for the detection of IPA on chest x-rays could be substantially increased. Although the increase in sensitivity was associated with a small decrease in specificity, ROC analysis indicated a significant improvement in overall performance. These findings are in agreement with previous studies which documented an improved detection performance of nodules or focal pneumonia [14]–[16]. Our results are noteworthy because they show that BSI is also supportive in a study group of highly variable imaging quality with respect to patient position and inspiration depth.

Whether and to which degree this improved detection performance changes patient management and would prompt earlier treatment can only be hypothesized at this point in time and any quantification is beyond the scope of this publication. Though multiple studies documented the limited sensitivity of CXR for the diagnosis of IPA [20]–[28], it still remains an important diagnostic procedure in these patients and appears not to necessarily trigger immediate CT imaging as documented by our study group acquired in 4 hospitals: the average time between CXR and CT was about 2.6 days. We do not know whether this delay is due to organizational reasons, a lack of immediate therapeutic consequences (patient was treated anyway) or the fact that a particularly subtle lesions had not been seen during the original readings. We believe that despite its limited sensitivity regarding IPA, or specificity regarding the potential causative organism, CXR has its role in the diagnostic work up of these delicate patients which might not be easily transferrable to CT in all situations. Application of this new processing technique has been shown to improve the detection of focal opacifications in radiographs, which in any case represents the more readily available imaging method.

The ideal situation would be to treat only patients suffering from IPA and to do this without any time delay [29]. In this scenario, imaging plays a crucial role. Current EORTC/MSG guidelines, especially the criteria for CT imaging, are very specific resulting in many non-classifiable cases. A broadening of the radiological criteria by including more non-specific findings would most likely result in an increased sensitivity for the detection of IPA, however, at the expense of a much lower specificity [30]. That way, imaging would be used as a tool to detect any morphological changes rather than revealing findings that indeed are suggestive for IPA.

Widespread nodules with a halo of ground glass in a peribronchovascular distribution have been described as the best discriminator for early IPA [31]. Prematurely obtained CT scans (e.g. immediately after onset of fever and without any radiographic changes) might demonstrate only non-specific findings harboring the risk for false negative interpretation or overdiagnosis, especially if assessed by less experienced radiologists. On the other hand, CT scans obtained in a later stage may provide diagnosis of IPA, but at the price of increased morbidity and mortality for the patient. One can hypothesize that a more sensitive chest radiographic technique may serve as trigger to obtain CT imaging without further delay, thus shortening the time to diagnosis. Meanwhile this CT acquisition, triggered by positive chest radiographic findings, will likely reveal findings indeed suggestive of IPA, consequently increasing its specific diagnostic yield. This hypothesis is supported by the fact that, according to our results, the largest benefit of BSI was seen for the subtle and very subtle cases. With the bone subtracted images, the number of detected very subtle cases quadrupled. Secondly we found a surprisingly long time delay between radiography and CT of 2.6 days on average for the diseased patients, showing a potential gain in time to diagnosis.

In our study, the baseline performance was higher for bedside images than for the upright images, which was not expected when considering the variable image quality and generally poorer condition of the patients undergoing bedside imaging. The superior baseline performance for the bedside images in our study was likely due to the fact that the group of bedside images contained relatively more obvious cases (group of obvious and *moderately* subtle cases) as compared to the upright group (55% versus 46%). Even so, we found a similar performance increase for both groups with an increase in AUC of 0.49 for upright images and of 0.40 for bedside images which failed to reach significance in the bedside group due to the smaller sample size.

Our study suffers from some limitations. Evaluation was performed separately for the right and left lung to prevent readers from being influenced by the contralateral findings and to be able to assess the performance for various types of lesions independently, with respect to conspicuity. From a clinical point of view this introduced a somewhat artificial component. Although readers did not know the percentage of diseased and normal lungs, they did know that prevalence was enriched which might have lowered their threshold to call opacifications positive. Lastly, the time difference between acquisition of the CXR and the CT does not exclude in this rapidly changing disease that extent and location of lesions differed between the two examinations. All radiographs were carefully evaluated by an experienced chest radiologist and the clinical researcher, confirming the radiographic findings with CT as reference standard. This issue was also considered less important given the fact that readers were asked to score the most obvious lesion per lung and not all lesions.

In conclusion, a new processing technique that digitally subtracts overlying bone structures in chest radiographs significantly improves the detection of fungal infections in chest radiographs. Shortcomings of CXR regarding its low sensitivity for the detection of IPA could be partly eliminated with the use of BSI, since we found an increase in sensitivity of 17%. Despite its inferiority to CT, CXR still represents an important role in the work up of febrile neutropenic patients, serving as a diagnostic screening procedure that is readily available, relatively easy to perform and providing immediate diagnostic information. Prospective studies will have to prove the impact of a substantially improved radiographic technique on the role of radiography in diagnosing IPA more promptly and triggering CT examinations for further morphological analysis.
